# Supplementary material for: A 350,000-year history of groundwater recharge in the southern Great Basin, USA
Source: Commun Earth Environ. 2023 Mar 31;4(1):98. doi: 10.1038/s43247-023-00762-0 (PMC11041671; doi:10.1038/s43247-023-00762-0)
Supplement: Supplementary file 1 — Supplemental material [file 43247_2023_762_MOESM1_ESM.pdf]

Supplementary Information for

A 350,000-year history of groundwater recharge in the southern Great Basin, USA

Tracie R. Jackson[1*], Simon D. Steidle[2*], Kathleen A. Wendt[3], Yuri Dublyansky[2], R. Lawrence Edwards[4], Christoph Spötl[2]
[1] *Nevada Water Science Center, U.S. Geological Survey, 500 Date Street, Boulder City, Nevada 89005, USA*
[2] *Institute of Geology, University of Innsbruck, Innrain 52, 6020 Innsbruck, Austria*
[3] *College of Earth, Ocean, and Atmospheric Sciences, Oregon State University, 101 SW 26th Street, Corvallis, Oregon 97330, USA*
[4] *School of Earth and Environmental Sciences, University of Minnesota, 116 Church Street SE, Minneapolis, MN 55455-0149, USA*
[*] *These authors contributed equally to this work*

Supplementary Note

This study, referred herein as the “Current Study”, uses the Death Valley version 3 steady-state (DV3-SS) groundwater-flow model developed by Halford and Jackson (2020). The groundwater-flow model conceptualization, construction, and calibration are documented in Halford and Jackson (2020), but also are summarized in this supplementary information. The groundwater-flow conceptualization was developed based on an understanding of the study area’s geology, hydrogeology, and modern predevelopment flow. DV3-SS model construction, including domain extent, discretization, and boundary conditions are described. The calibration methodology is summarized to explain estimation of spatially distributed recharge and hydraulic properties. Modifications to the published DV3-SS model for the Current Study are described in detail. The modified DV3-SS model is published in a separate data release (Jackson, 2022).

The study area for the Current Study and the DV3 study are different because of different study objectives. The Current Study focuses on estimating contributing recharge areas to Devils Hole, based on different climatic conditions, and includes the Ash Meadows and Alkali Flat–Furnace Creek Ranch (AFFCR) groundwater basins. The DV3-SS model focused on predevelopment flow, groundwater development, and radionuclide transport within the Ash Meadows, AFFCR, Pahute Mesa–Oasis Valley (PMOV), and Pahrump to Death Valley South (PDVS) groundwater basins (Sup. fig. 1).

The Current Study includes Ash Meadows and AFFCR groundwater basins because Halford and Jackson (2020) concluded that these basins are coupled and are better considered as a single groundwater basin. The basins are hydraulically connected by permeable basin-fill and carbonate rocks within a 3.2 to 8 km wide corridor north of Devils Hole (Sup. fig. 2). Estimated predevelopment groundwater flow through the corridor is about 3.15 x 10^6 m^3/yr.

The PDVS groundwater basin was excluded from the Current Study because the basin is hydraulically isolated from the Ash Meadows and AFFCR groundwater basins. Low-permeability siliciclastic rocks crop out in the Spring Mountains along the southern boundary of the Ash Meadows groundwater basin between Pahrump Valley and Devils Hole (Sweetkind et al., 2010). The distribution of these low-permeability rocks is controlled by a thrust fault. Low-permeability rocks in the upper thrust plate dip westward and northwestward, impeding northward and westward groundwater movement from carbonate rocks in the lower plate toward Devils Hole and Ash Meadows discharge area (Winograd and Thordarson, 1975). Large hydraulic gradients (Fenelon et al., 2016, plate 1) and geochemically distinct groundwaters (Winograd and Thordarson, 1975) between Devils Hole and Pahrump Valley provide hydrologic evidence that siliciclastic rocks in the Spring Mountains form a hydraulic barrier to groundwater flow.

The PMOV groundwater basin was excluded from the Current Study because the basin largely is hydraulically isolated from the neighboring Ash Meadows and AFFCR groundwater basins. Basin budget analyses (Fenelon et al., 2016; Jackson et al., 2021) and groundwater modeling of PMOV basin boundaries (Fenelon et al., 2016; Halford and Jackson, 2020) indicate that negligible interbasin flow occurs between the PMOV–Ash Meadows boundary (less than 0.1% of Ash Meadows water budget), and insignificant interbasin flow occurs between the PMOV–AFFCR boundary (less than 2% of AFFCR water budget).

Expanding the study area of the Current Study to include the PMOV basin or other bounding basins would have no effect on the water levels in Devils Hole. Devils Hole water levels will be unaffected by potential, but likely insignificant, interbasin flows into the study area along basin boundaries distant from Devils Hole (Fenelon et al., 2016; Halford and Jackson, 2020; Jackson et al., 2021). During wetter periods, groundwater-flow paths shorten between recharge and discharge areas, and more discharge areas form. Therefore, the contributing area to Devils Hole will be smaller. During drier periods, significant interbasin flows are unlikely across basin boundaries because modern discharge areas will either decrease in areal extent, remain the same size but have decreased discharge rates, or disappear due to lowered water levels. Therefore, inclusion of the Ash Meadows and AFFCR basins is adequate for estimating recharge volumes under different climate scenarios.

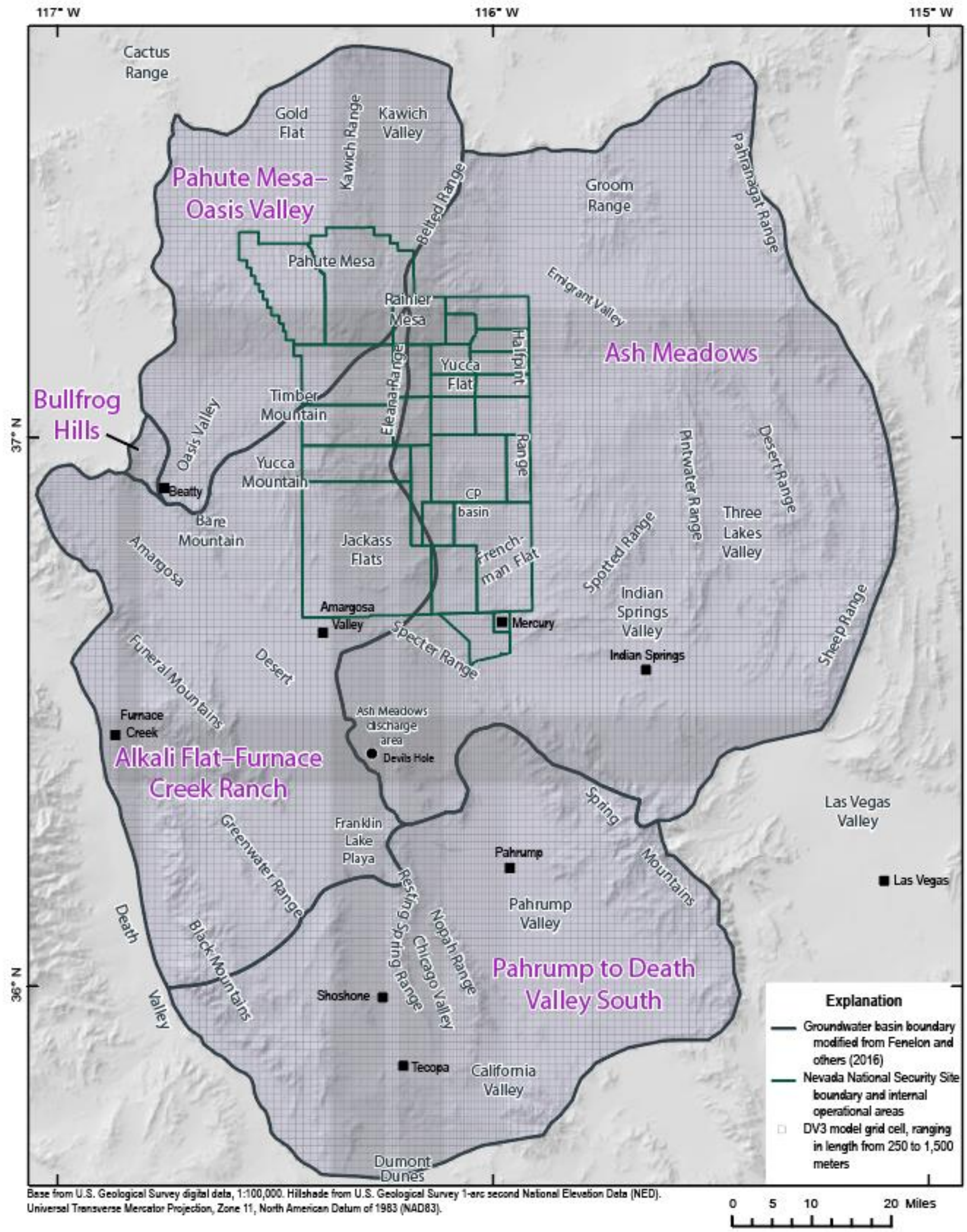

Supplementary figure 1. DV3-SS model domain extent, spatial discretization, and physiographic features.

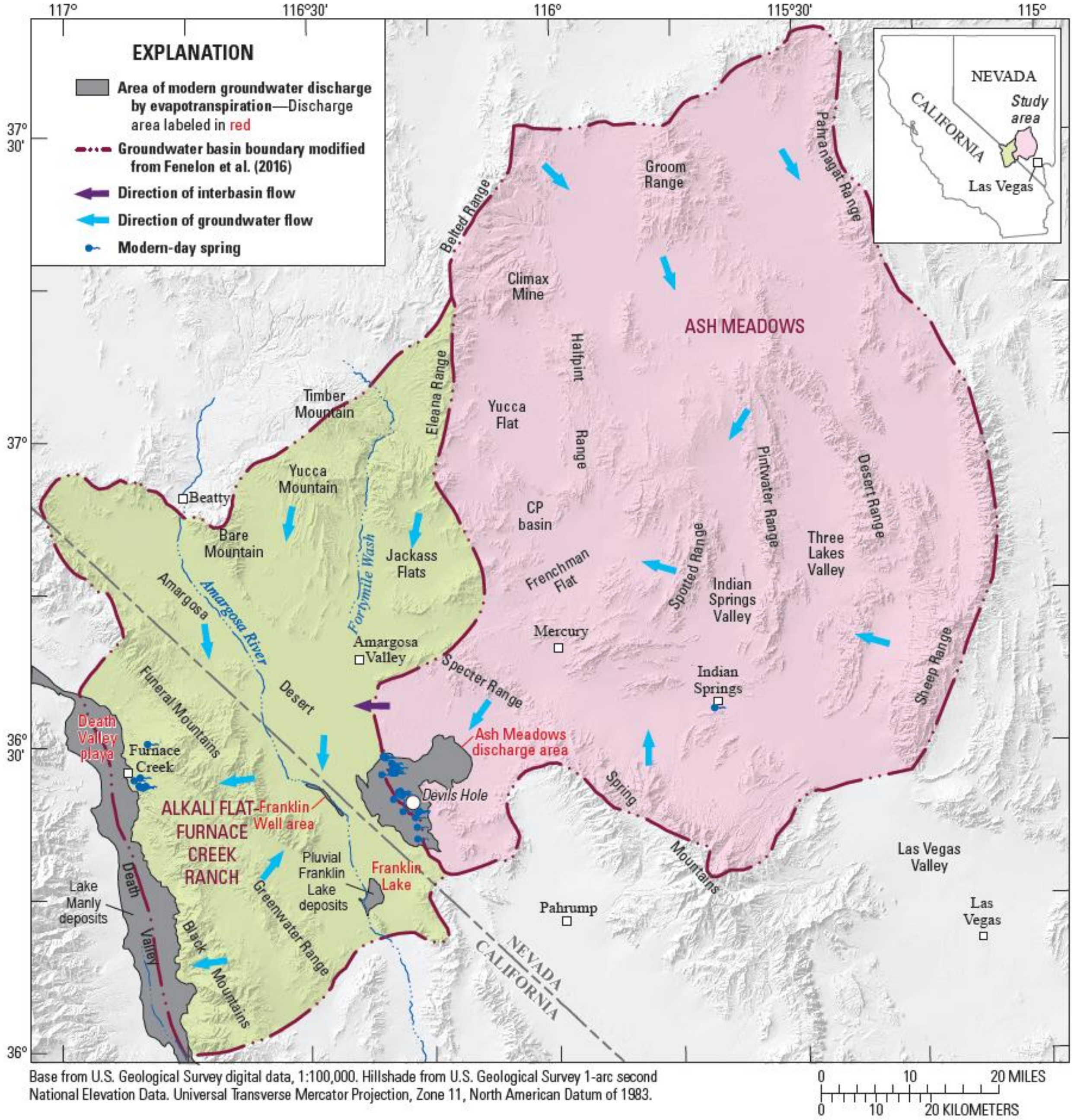

Supplementary figure 2. Extent of modified DV3-SS model, as used in the Current Study.

Geologic units were formed by magmatic activity, volcanism, and depositional processes in the study area. These rocks subsequently have been uplifted, thrusted, offset, and deformed. Depositional, magmatic, volcanic, and tectonic processes have juxtaposed geologic units into a complex three-dimensional framework that affects groundwater flow. The geologic history is divided into six periods of deposition and tectonic deformation: (1) Precambrian and Paleozoic marine and non-marine deposition; (2) Late Paleozoic to Early Cenozoic folding, thrusting, and strike-slip faulting; (3) Mesozoic igneous intrusion; (4) Cenozoic volcanism; (5) Cenozoic normal and strike-slip faulting; and (6) Cenozoic basin-fill deposition (Sup. table 1).

Precambrian and Paleozoic marine and orogenic processes resulted in two major sedimentation sequences of siliciclastic and carbonate rocks (Winograd and Thordarson, 1975; Laczniak et al., 1996; Slate et al., 2000; Sweetkind et al., 2010). Marine sedimentary rocks, largely siltstones and sandstones (altered to quartzites), were deposited from Neoproterozoic to Lower Cambrian (Sup. table 1), where deposits thicken westward in the study area. The siliciclastic rocks are overlain by thick sequences of Middle Cambrian to Devonian carbonate rocks. From the Late Devonian to Mississippian, siliciclastic rocks, predominantly shales, argillites, and sandstones (altered to quartzites) interrupted marine carbonate deposition in the eastern part of the study area. Siliciclastic sediments were derived from uplifted areas north of the study area (Sweetkind et al., 2010). Marine carbonate deposition continued from the Late Mississippian to Permian (Sup. table 1).

Supplementary table 1. Major lithology and occurrence descriptions of hydrogeologic units in the Current Study area.

[Abbrev., Abbreviation; AM, Ash Meadows groundwater basin; AFFCR, Alkali Flat-Furnace Creek Ranch groundwater basin; CDVVF, Central Death Valley volcanic field; SWNVF, southwestern Nevada volcanic field. Table summarizes information obtained from Noble et al. (1964); Noble et al. (1984); Blankennagel and Weir (1973); Winograd and Thordarson (1975); Byers et al. (1976); Christiansen et al. (1977); Simonds (1989); Sawyer et al. (1994); Laczniak et al. (1996); Prothro and Drellack (1997); Slate et al. (2000); Sweetkind et al. (2010); Taylor and Sweetkind (2014).]

Era	Period	Abbrev.	Hydrogeologic unit	Major lithology	Occurrence
**Cenozoic basin-fill and volcanic sediments (unconsolidated)**					
Cenozoic	Tertiary - Quaternary	YAA	Younger alluvial aquifer	Coarse-grained alluvium and colluvium with minor debris-flow and eolian sediments. Proximal to distal fan alluvial deposits grade from gravels and sands to silts and clays, whereas fluvial deposits consist of gravel and sand. Debris flows are poorly sorted coarse- and fine-grained deposits. Eolian deposits are well-sorted dunal sands or sand sheets.	Saturated basin fill beneath ephemeral channels, such as Amargosa River and Fortymile Wash, and beneath depositional basins, such as Death Valley, Amargosa Desert, and Crater, Yucca, and Frenchman Flats [AFFCR, AM].
		OAA	Older alluvial aquifer		
		YACU	Younger alluvial confining unit	Fine-grained playa and lacustrine deposits. Deposits consist of alternating sequences of fine sand, silt, and clay. Playa sediments are imbued with salt and capped by a salt crust.	Saturated basin-axis and playa deposits beneath valley floors, such as Death Valley, Amargosa Desert, and Yucca and Frenchman Flats [AFFCR, AM].
		OACU	Older alluvial confining unit		
		LA	Limestone aquifer	Lacustrine and spring deposits of limestone and travertine.	Localized saturated units in Amargosa Desert [AFFCR, AM].
		LFU	Lava-flow unit	Localized rhyolitic and basaltic lava flows, cinder cones, and nonwelded tuff. Rocks erupted from CDVVF.	Unit typically located above water table, except for localized lava flows in Amargosa Desert and Crater Flat [AFFCR].
	Tertiary	YVU	Younger volcanic-rock unit	Localized volcanic tuffs.	Unit typically located above water table, except for localized tuffs in Desert Range [AM].
		SmAA	Coarse-grained units	Coarse-grained (gravel and sand) basin-fill deposits, basaltic lava flows, and limestones.	Saturated basin fill beneath Amargosa Desert, Crater and Jackass Flats, and the Ash Meadows discharge area [AFFCR, AM].
		SmCU	Fine-grained units	Fine-grained (silt and clay) basin-fill deposits.	Saturated basin fill beneath Amargosa Desert, Crater and Jackass Flats, and the Ash Meadows discharge area [AFFCR, AM].

Supplementary table 1. Continued.

Era	Period	Abbrev.	Hydrogeologic unit	Major lithology	Occurrence
**Cenozoic volcanic rocks of the southwestern Nevada volcanic field**					
Cenozoic	Tertiary	TMVA	Thirsty Canyon-Timber Mountain volcanic-rock aquifer	Variably welded ash-flow tuffs, ash-fall tuffs, rhyolite and trachyte lavas and domes, and intracaldera landslide breccia with minor nonwelded tuff.	Volcanics erupted from Timber Mountain caldera complex. Unit saturated beneath Yucca Mountain, and Jackass, Yucca, and Frenchman Flats [AFFCR, AM].
		PVA	Paintbrush volcanic-rock aquifer	Rhyolitic lavas, variably welded ash-flow tuffs, nonwelded ash-fall tuff, bedded tuff, and fluvially reworked tuff. Nonwelded tuffs have argillic, hematitic, silicic, or zeolitic alteration.	Volcanics erupted from the Claim Canyon caldera. Unit saturated beneath Yucca Mountain, and Jackass, Yucca, and Frenchman Flats [AFFCR, AM].
		CHVU	Calico Hills volcanic-rock unit	Variably welded ash-flow tuffs, nonwelded zeolitically altered ash-fall and bedded tuffs, rhyolitic lavas, and volcaniclastic deposits.	Volcanics erupted from the Silent Canyon caldera complex. Unit saturated beneath Yucca Mountain, and Jackass and Yucca Flats [AFFCR, AM].
		WVU	Wahmonie volcanic-rock unit	Biotite-rich nonwelded tuff, andesitic-to-dacitic lavas, tuffaceous sandstone, and tuff breccia.	Volcanics erupted from stratovolcanoes in the Wahmonie volcanic center near Skull Mountain. Unit saturated beneath Jackass and Yucca Flats [AFFCR, AM].
		CFPPA	Crater Flat–Prow Pass aquifer	Rhyolitic-to-andesitic lava flows and variably welded ash-flow tuffs. Partly welded to nonwelded tuffs are commonly zeolitized.	Location of source for erupted volcanics unknown. Unit saturated beneath Rainier Mesa, Yucca Mountain, and Yucca Flat [AFFCR, AM].
		CFBCU	Crater Flat–Bullfrog confining unit	Nonwelded and variably welded ash-flow and ash-fall tuffs. Nonwelded tuff commonly zeolitized.	Volcanics erupted from the Silent Canyon caldera complex. Unit saturated beneath Yucca Mountain [AFFCR].
		CFTA	Crater Flat–Tram aquifer	Nonwelded to variably welded ash-flow tuffs. Mostly partially welded to nonwelded tuffs with localized areas of dense welding.	Volcanics possibly erupted from an unnamed caldera beneath northern Crater Flat. Unit saturated beneath Yucca Mountain [AFFCR].
		BRU	Belted Range unit	Rhyolitic lavas and densely welded ash-flow tuffs with minor nonwelded (commonly zeolitized) tuff.	Volcanics erupted from Silent Canyon caldera complex. Unit saturated beneath Yucca Flat [AM].
		OVU	Older volcanic-rock unit	Undifferentiated older volcanic rocks of the SWNVF, including lavas, variably welded ash-flow tuffs, zeolitically altered nonwelded tuffs, reworked tuff, and volcaniclastic rocks.	Volcanic rocks erupted from volcanic centers either in or north of the SWNVF. Unit saturated beneath Rainier Mesa, Yucca Mountain, and Yucca and Frenchman Flats [AFFCR, AM].
		VSU upper	Upper volcanic-sedimentary rock unit	Undifferentiated volcanic and sedimentary rocks, including welded and nonwelded tuffs, rhyolitic-to-basaltic lava flows, alluvium, colluvium, and eolian, fluvial, and lacustrine deposits. Rocks not associated with previously defined volcanic and sedimentary units.	VSU upper and VSU lower contain rocks that overlie and underlie volcanic rocks of the SWNVF, respectively. Outside the SWNVF boundary, the division between VSU upper and VSU lower is arbitrary. Saturated unit is regionally extensive throughout study area [AFFCR, AM].
		VSU lower	Lower volcanic-sedimentary rock unit		

Supplementary table 1. Continued

Era	Period	Abbrev.	Hydrogeologic unit	Major lithology	Occurrence
			**Pre-Cenozoic rocks**		
Mesozoic - Cenozoic		ICU	Intrusive-rock confining unit	Mesozoic granite, granodiorite, quartz monzonite, and tonalite in stocks, dikes, and sills. Cenozoic (Oligocene and Miocene) granite, gabbro, and diorite plutons associated with extension and caldera-related volcanism.	Localized stocks in Greenwater Range, and north of Rainier Mesa and Yucca Flat [AFFCR, AM].
Paleozoic	Mississippian - Permian	UCA	Upper carbonate-rock aquifer	Limestone, dolomite, and calcareous shale.	Localized saturated unit above UCCU in western Yucca Flat [AM].
	Devonian - Mississippian	UCCU	Upper clastic-rock confining unit	Quartzite, argillite, conglomerate, sandstone, siltstone, and shale with minor limestone. Carbonate and siliciclastic rocks were derived from debris flows and turbidites, where sediments grade from coarse to fine from ridges to the valley floor.	Localized unit saturated beneath northern Jackass Flats and western and northern Yucca Flat [AFFCR, AM].
	Cambrian - Devonian	LCA_T1	Lower carbonate-rock aquifer (thrusted)	Massive dolomite and limestone with minor quartzite, chert, shale and calcareous siltstone. LCA includes Mississippian and Pennsylvanian carbonate rocks, where these rocks are not separated by UCCU.	Saturated unit occurs beneath the Spring Mountains, Sheep Range, Specter Range, Skull Mountain, and central Yucca Flat [AFFCR, AM].
		LCA	Lower carbonate-rock aquifer	Massive dolomite and limestone with minor quartzite, chert, shale and calcareous siltstone. LCA includes Mississippian and Pennsylvanian carbonate rocks, where these rocks are not separated by UCCU.	Saturated unit is regionally extensive throughout AM groundwater basin, occurs beneath Amargosa Desert, and Spring and Funeral Mountains [AFFCR, AM].
		LCCU_T1	Lower clastic-rock confining unit (thrusted)	Quartzite, sandstone, siltstone, shale, minor limestone and dolomite, and their metamorphic equivalents.	Saturated unit occurs beneath the Spring Mountains, Sheep Range, Specter Range, Funeral Mountains, and Rainier Mesa [AFFCR, AM].
Neo-Proterozoic to Cambrian		LCCU	Lower clastic-rock confining unit	Quartzite, sandstone, siltstone, shale, minor limestone and dolomite, and their metamorphic equivalents.	Saturated unit occurs throughout study area [AFFCR, AM].
Pre-Cambrian		XCU	Crystalline-rock confining unit	Metamorphosed sedimentary rocks, granite, gneiss, and quartzfeldspathic schist.	Saturated unit occurs beneath Funeral and Black Mountains [AFFCR].

Precambrian and Paleozoic rocks were subjected to compressional tectonic forces from the Late Paleozoic to Early Cenozoic. Siliciclastic and carbonate rocks were offset by regional thrust faulting, which emplaced Neoproterozoic to Lower Cambrian siliciclastic rocks over younger Paleozoic rocks (Sweetkind et al., 2010). One example is the thrust faulting that occurred in the Spring Mountains that formed a hydraulic barrier between Devils Hole and Pahrump Valley (Winograd and Thordarson, 1975). Many mountain ranges have exposed thrust faults, including the Belted, Eleana, and Specter Ranges as well as Bare Mountain, Spring Mountains, and Funeral Mountains (Sweetkind et al., 2010). Siliciclastic and carbonate rocks also were subjected to folding and strike-slip faulting (Laczniak et al., 1996). Compressional tectonics have formed broad anticlines and synclines in the Precambrian and Paleozoic sequence within the Ash Meadows groundwater basin. Mesozoic rocks are largely absent due to uplift and erosion of Precambrian and Paleozoic rocks (Sweetkind et al., 2010). Therefore, Mesozoic rocks are minor and occur as localized granitic intrusions (Sup. table 1).

Volcanic and associated sedimentary rocks formed during the Cenozoic (Sup. table 1). From the Oligocene to Miocene, thick sequences of volcanic rocks were deposited by caldera-forming eruptions in the southwestern Nevada volcanic field, located in the northern part of the AFFCR groundwater basin (Byers et al., 1976*;* Christiansen et al., 1977*;* Sawyer et al., 1994*;* Laczniak et al., 1996*;* Prothro and Drellack, 1997). Volcanic centers within the southwestern Nevada volcanic field caldera complex erupted rhyolitic-to-dacitic lava flows, variably welded ash-flow tuffs, ash-fall tuffs, and volcaniclastic rocks (Winograd and Thordarson, 1975*;* Laczniak et al., 1996*;* Sweetkind et al., 2010). From the Miocene to Pliocene, localized rhyolitic and basaltic lava flows erupted from the central Death Valley volcanic field near the Greenwater Range

(Sweetkind et al., 2010). During the Holocene, localized basaltic lava flows erupted from five cinder cones in the Crater Flat volcanic field (Wells et al., 1990).

Large-scale normal faulting occurred during and after the period of Cenozoic volcanism (Winograd and Thordarson, 1975; Laczniak et al., 1996; Prothro and Drellack, 1997; Sweetkind et al., 2010). From the Mid-Tertiary to Quaternary, normal faults formed the Basin and Range topography of the Great Basin province. Normal faults are the most common structural feature in the study area and displacement along normal faults continues today (Winograd and Thordarson, 1975). Normal faults typically have displaced the Precambrian through Cenozoic section by less than 150 m; however, in some cases, normal faults have displaced rocks more than 300 m (Winograd and Thordarson, 1975). Sedimentary rocks associated with volcanism occur throughout the study area, and consist of tuffaceous sandstone and siltstone, lacustrine tuff, and claystone (Sup. table 1).

Cenozoic unconsolidated basin fill occurs throughout the study area in intermontane basins (Sup. table 1; Sweetkind et al., 2010; Taylor and Sweetkind, 2014). Coarse-grained basin fill typically consists of alluvial and colluvial deposits with minor fluvial, debris-flow, and eolian deposits. Fine-grained basin fill consists of playa and lacustrine sediments. Limestone and spring deposits also are present locally.

Previous investigators have classified geologic units into hydrogeologic units based on rock hydraulic properties (Sup. table 1; Winograd and Thordarson, 1975; Laczniak et al., 1996; Slate et al., 2000; Bechtel Nevada, 2006; Fenelon et al., 2010; 2012; 2016). Hydrogeologic units categorize geologic units as aquifers or confining units based on their ability to store and transmit groundwater. Halford and Jackson (2020) developed a simplified hydrogeologic framework model

(DV3-HFM) that groups previously defined hydrogeologic units into four broad categories: carbonate rocks, volcanic rocks, basin fill, and low-permeability rocks (Sup. table 2).

Supplementary table 2. Relation between simplified rock types in Death Valley (version 3) hydrogeologic framework model and previously defined hydrogeologic units (see Table 1) in the study area.

DV3-HFM Rock Type	Hydrogeologic unit abbreviation	Hydrogeologic unit
Carbonate Rocks	UCA	Upper carbonate-rock aquifer
	LCA_T1	Lower carbonate-rock aquifer (thrusted)
	LCA	Lower carbonate-rock aquifer
Volcanic Rocks	LFU	Lava-flow unit
	YVU	Younger volcanic-rock unit
	TMVA	Thirsty Canyon-Timber Mountain volcanic-rock aquifer
	PVA	Paintbrush volcanic-rock aquifer
	CHVU	Calico Hills volcanic-rock unit
	WVU	Wahmonie volcanic-rock unit
	CFPPA	Crater Flat–Prow Pass aquifer
	CFBCU	Crater Flat–Bullfrog confining unit
	CFTA	Crater Flat–Tram aquifer
	BRU	Belted Range unit
Basin Fill	YAA	Younger alluvial aquifer
	YACU	Younger alluvial confining unit
	OAA	Older alluvial aquifer
	OACU	Older alluvial confining unit
	LA	Limestone aquifer
	VSU upper	Upper volcanic-sedimentary rock unit
Low-Permeability Rocks	OVU	Older volcanic-rock unit
	VSU lower	Lower volcanic-sedimentary rock unit
	ICU	Intrusive-rock confining unit
	UCCU	Upper clastic-rock confining unit
	LCCU_T1	Lower clastic-rock confining unit (thrusted)
	LCCU	Lower clastic-rock confining unit
	XCU	Crystalline-rock confining unit

Halford and Jackson (2020) grouped hydrogeologic units into four broad categories based on a detailed analysis that related aquifer-test transmissivity estimates to rock type. Analysis results indicated that only four broad categories of rock type can be related to transmissivity:

carbonate rocks, volcanic rocks, basin fill, and low-permeability rocks. Carbonate rocks were not further subdivided because few wells penetrate the carbonate aquifer. Volcanic rocks were not subdivided because distributions of log-hydraulic conductivity were similar between younger volcanic and Tertiary volcanic units. Probability distributions overlapped considerably between the Tertiary volcanic unit that had been differentiated into eight hydrogeologic units (Belcher et al., 2002; p. 12). Log-hydraulic conductivity distributions of alluvial aquifer and alluvial confining unit differed little; therefore, units were grouped into a single category of basin fill. Low-permeability rocks consist of granitic rocks, siliciclastic rocks, and older volcanic rocks, which form the basement of the study area. Low-permeability rocks were not further subdivided because there were only a small number of transmissivity estimates. Heterogeneity within hydrogeologic units was simulated in the DV3-SS model using a pilot-point approach, which is discussed in the "*Supplemetary Methods*" section.

Groundwater recharge occurs where precipitation infiltrates permeable rocks and percolates below the root zone to the water table, either through an interconnected network of fractures or the rock matrix. Greater amounts of precipitation occur at higher altitudes; consequently, greater amounts of recharge typically occur in highland areas. An exception to this conceptualization occurs where low-permeability rocks underlie highland areas. In these areas, precipitation from snowmelt (or high-intensity rainfall) flows downgradient and infiltrates into adjacent alluvial-fan deposits. Some surface runoff, and subsequent recharge, occurs in ephemeral channels draining highland areas.

Potential groundwater recharge for most of the Ash Meadows groundwater basin occurs in highland areas such as the Spring Mountains, and the Belted, Groom, Pahranagat, Sheep, Desert, Pintwater, and Spotted Ranges (Sup. fig. 2; Fenelon et al., 2016). Potential groundwater recharge

in the AFFCR groundwater basin mostly occurs in the highland areas of Yucca and Timber Mountain.

Groundwater moves from highland recharge areas to areas where groundwater discharges predominantly at springs and seeps. Groundwater generally moves in a south-southwest direction in Ash Meadows groundwater basin toward Ash Meadows discharge area, which is the principal discharge area (Sup. fig. 2; Fenelon et al., 2016). An additional amount of groundwater moves westward as interbasin flow from the Ash Meadows groundwater basin into the AFFCR groundwater basin (Sup. fig. 2). This interbasin flow ultimately discharges from springs, seeps, and phreatophytes in the Furnace Creek area of Death Valley, which is the principal discharge area in the AFFCR groundwater basin (Sup. fig. 2). Groundwater within recharge areas in the AFFCR basin generally moves southwestward into the Amargosa Desert and then westward toward the Furnace Creek discharge area.

Supplementary Methods

The DV3-SS model (Halford and Jackson, 2020) was developed to simulate modern predevelopment groundwater flow, where modern conditions are defined as occurring within the last 100 years. The DV3-SS model is a three-dimensional, finite-difference, numerical model (MODFLOW 2005; Harbaugh, 2005). The DV3-SS model domain includes the Ash Meadows, AFFCR, PMOV, and PDVS groundwater basins. However, since the Current Study truncated the model domain of the DV3-SS model to include only the Ash Meadows and AFFCR basins, the DV3-SS model description is limited to these two basins.

Recharge and hydraulic-conductivity distributions in the Ash Meadows and AFFCR groundwater basins were estimated by simultaneously calibrating three, three-dimensional, numerical groundwater models of the study area (Halford and Jackson, 2020). Multiple models were used to differentiate, and better interpret, effects of predevelopment flow, groundwater development, and aquifer tests on the groundwater system. Multiple models allow boundary conditions to be better constrained to known (predevelopment or pumping) conditions and allow comparison of simulated responses to those known conditions. For example, discharge from *Big Spring* in the Ash Meadows discharge area averaged 0.065 m^3/s prior to development and the pool altitude was 683 m. Multiple models allowed simulating *Big Spring* as a specified discharge of 0.065 m^3/s prior to development and as a capture-limited, head-dependent boundary during groundwater development. This prevented misfits between simulated and measured pool altitudes from affecting simulated capture during groundwater development.

Predevelopment conditions, the *ER-6-1-2 main* multiple-well aquifer test (MWAT), and groundwater development in Ash Meadows and AFFCR groundwater basins were simulated with three separate, but coupled, groundwater-flow models. The DV3-SS model simulated modern

predevelopment conditions and informed estimates of recharge rates and hydraulic conductivities. The DV3-ER612 model simulated changes from pumping during *ER-6-1-2 main* well development and aquifer testing from February 5–July 23, 2004, and informed estimates of hydraulic properties in Yucca Flat (Sup. fig. 2). The DV3-AM-AFFCR model simulated water-level changes and spring captures from groundwater development between 1950 and 2018 in the Ash Meadows and AFFCR groundwater basins. Transient DV3-ER612 and DV3-AM-AFFCR models informed estimates of hydraulic conductivity, specific yield, and specific storage where pumping measurably changed water levels or discharges. See Halford and Jackson (2020) for details on the transient DV3-ER612 and DV3-AM-AFFCR models.

The DV3-SS model, and all other groundwater-flow models developed by Halford and Jackson (2020), shared a common domain, grid, hydrogeologic framework, and hydraulic properties. Model domains extended laterally to no-flow boundaries at the outer boundaries of the four groundwater basins (Sup. fig. 1). Each model was divided areally into 268 rows of 221 columns of variably spaced, rectangular cells that ranged from 250-m to 1500-m on a side. Furnace Creek area, Ash Meadows discharge area, Rainier Mesa, and Yucca Flat were areas of finer discretization (Sup. fig. 1). The model grid extended vertically from the water table to 1,000 m below sea level and was divided into six layers that successively increased in thickness with depth. Layer 1 was 0.3-m thick to better simulate groundwater and surface-water interaction, and drainage at the water table. All models have the same DV3 hydrogeologic framework (Sup. fig. 3; Halford and Jackson, 2020). This framework has nine hydrogeologic units that were divided primarily based on rock type and depth below the water table (Sup. table 3). Hydraulic-property distributions are identical in each calibrated model because all models share a common hydrogeologic framework.

Supplementary table 3. Lithology and occurrence descriptions of hydrogeologic units in the DV3 model within the Ash Meadows and AFFCR groundwater basins.

[AM, Ash Meadows groundwater basin; AFFCR, Alkali Flat-Furnace Creek Ranch groundwater basin].

Rock Type	DV3 hydrogeologic unit	Description	Hydrogeologic units from Table 1
**Carbonate Rocks**	Shallow, low-permeability carbonate rocks	Occur primarily beneath Rainier Mesa, western Yucca Flat, Specter Range, and Sheep Range.	UCA, LCA_T1
	Shallow carbonate rocks	Regionally extensive in the AM basin. Occurs in the southern Funeral Mountains in the AFFCR basin.	LCA_T1, LCA
	Deep carbonate rocks	Regionally extensive in the AM basin. Occurs beneath Shoshone Mountain, Yucca Mountain, Jackass Flats, Amargosa Desert, and the Funeral Mountains in the AFFCR basin.	LCA_T1, LCA
**Volcanic Rocks**	Shallow volcanic rocks	Occurs beneath Timber Mountain, Yucca Mountain, and Jackass Flats in AFFCR basin. Forms localized units beneath Yucca Flat, Frenchman Flat, and Emigrant Valley in the AM basin.	LFU, YVU, TMVA, PVA, CHVU, WVU, CFPPA, CFBCU, CFTA, BRU
**Basin Fill**	Shallow basin fill	Upper 60 m of saturated basin fill beneath valleys, including the Amargosa Desert, Death Valley, Emigrant Valley, Yucca Flat, and Frenchman Flat.	YAA, YACU, OAA, OACU, LA, VSU upper
	Deep basin fill	Remainder of saturated basin fill beneath valleys, including the Amargosa Desert, Death Valley, Emigrant Valley, Yucca Flat, and Frenchman Flat.	YAA, YACU, OAA, OACU, LA, VSU upper
**Volcanic-Sedimentary Rocks**	Volcanic-sedimentary rocks	Shallow volcanic-sedimentary rocks. Rocks form the low-permeability "tuff pile" beneath Rainier Mesa, Yucca Flat, and Frenchman Flat, and extend beneath Emigrant Valley in the AM basin. Rocks extend from Amargosa Desert to Greenwater Range in AFFCR basin.	OVU, VSU upper
**Low-Permeability Rocks**	Deep volcanic rocks	Deep volcanic rocks and volcanic-sedimentary rocks. Occur beneath Yucca Mountain, Amargosa Desert, Death Valley, and Eagle Mountain in AFFCR basin. Saturated rocks are localized beneath Emigrant Valley in the AM basin.	TMVA, PVA, CHVU, WVU, CFPPA, CFBCU, CFTA, BRU, OVU, VSU lower
	Granitic and siliciclastic rocks	Clastic sediments (and their metamorphosed equivalents), and granite, granodiorite, quartz monzonite, and tonalite in stocks, dikes, sills, and plutons. Rocks extend between Pahrump Valley and the Ash Meadows discharge area. Rocks occur beneath the Greenwater Range, Black Mountains, and northern Funeral Mountains in the AFFCR basin. Rocks are saturated to the west, north, and east of Yucca Flat in the Eleana Range, Climax Stock, and Halfpint Range, respectively.	ICU, SCU, UCCU, LCCU_T1, LCCU, XCU

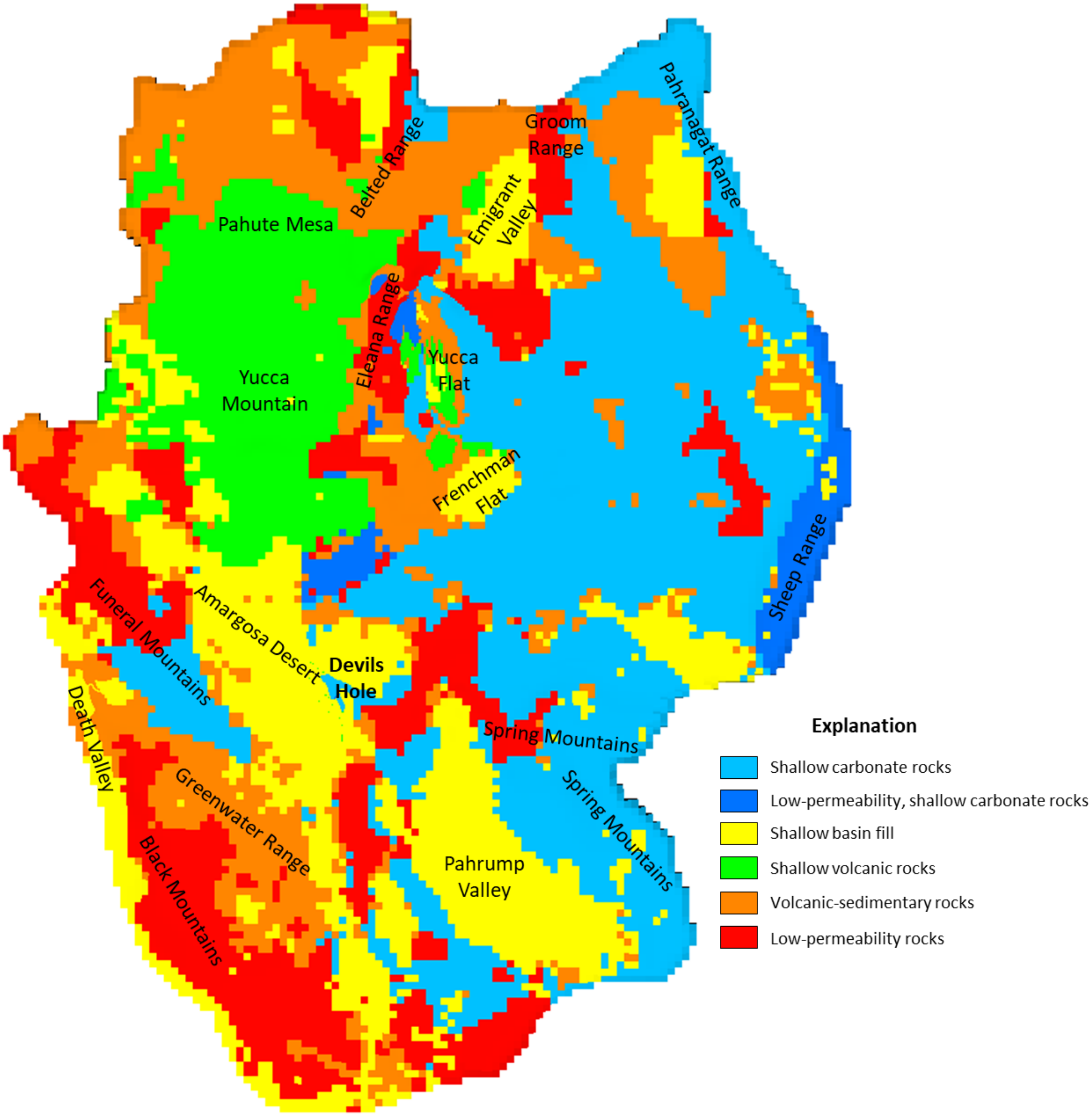

Supplementary figure 3. DV3 hydrogeologic framework in DV3-SS model showing hydrogeologic units occurring at the water table.

The outer edges of the DV3 study area (Sup. fig. 4) and the bottom of layer 6 at 1,000 m below sea level were no-flow boundaries in all groundwater models. Internal boundaries between groundwater basins were not simulated explicitly as no-flow boundaries. Instead, hydrogeologic units formed hydraulic barriers or conduits to groundwater flow based on estimated hydraulic properties (Sup. fig. 3). The top surface of each groundwater model was the water table. The DV3-SS model simulated recharge to the top surface of layer 1 across the entire model domain.

More than 96% of the predevelopment discharge was specified in the DV3-SS model (Sup. fig. 4) using the well package in MODFLOW (Harbaugh, 2005). Specified discharges from springs totaled 3.7×10^7 m^3/yr as measured, where 18 of 27 springs were in Ash Meadows discharge area (Sup. fig. 4). The remaining specified discharge was evapotranspired discharge in Ash Meadows discharge area, Furnace Creek area, Franklin Well area, Oasis Valley, Pahrump Valley, and Lower Amargosa area, which were simulated as specified discharges from layer 1 (Sup. fig. 4).

Remaining discharges in evapotranspiration areas not simulated as specified discharges were simulated as specified heads (Sup. fig. 4) because discharge estimates were uncertain and reference water levels were needed. Discharges from Franklin Lake and Death Valley playa were uncertain relative to water-table altitudes and hydraulic conductivities in these areas.

Heterogeneous hydraulic properties and recharge rates were distributed using pilot points (RamaRao et al., 1995). Hydraulic properties smoothly varied within hydrogeologic units but were not extrapolated between hydrogeologic units. Recharge similarly was distributed using pilot points, but preferred rates were guided by a conceptual model that considered water availability (precipitation) and occurrence of low-permeability rocks at the water table that likely impede infiltration.

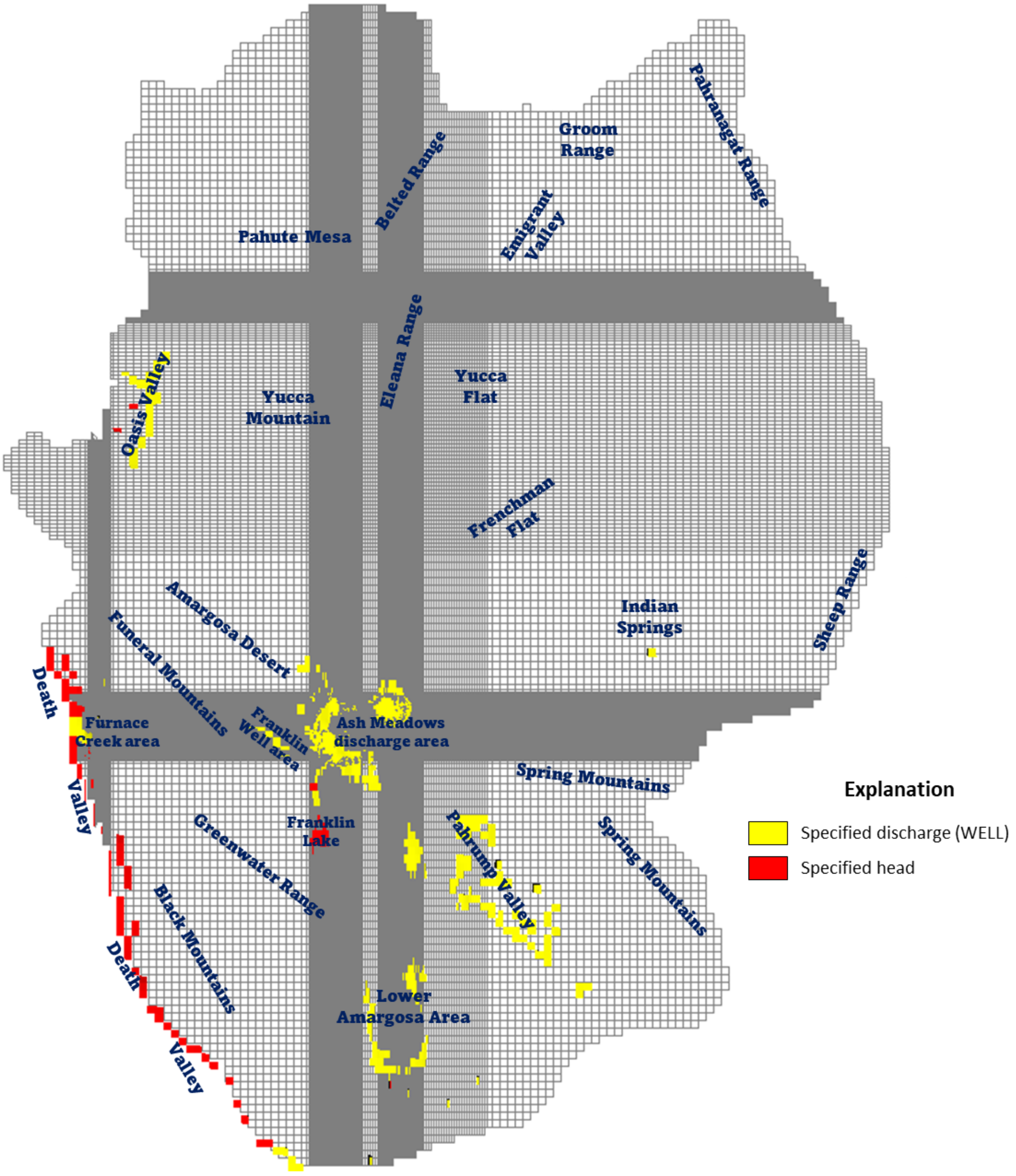

Supplementary figure 4. Boundary conditions in DV3-SS model.

Hydraulic properties and recharge rates were assigned to pilot points and adjusted to minimize a weighted sum-of-squares objective function with the parameter estimation program PEST (Doherty, 2010). Measured observations were compared to simulated equivalents in the objective function. Measured observations in the DV3-SS model included water-level altitudes in wells, water-level differences between paired wells, transmissivity estimates from aquifer tests, spring-pool altitudes, water-table altitudes in evapotranspiration areas, and water-table altitude comparisons to land surface. Conceptual distributions of recharge and hydraulic properties were incorporated in the objective function through Tikhonov regularization and primarily influenced estimates where data were limited. See Halford and Jackson (2020) for extensive calibration details.

Simulated and measured steady-state water levels in the DV3-SS model agreed with average and root-mean-square (RMS) errors of +0.3 m and 15 m, respectively. These differences are not large relative to the 2,682-m range of measured water levels. Measured water-level altitudes range from -85 to 2,592 m in Death Valley and the Spring Mountains, respectively. Steady-state water-level residuals show little spatial pattern of significance, suggesting a good overall fit between simulated and measured water levels. See Halford and Jackson (2020) for an extensive discussion of model calibration results.

The DV3-SS model domain was truncated (Halford and Jackson, 2020) in the Current Study to the Ash Meadows and AFFCR groundwater basins (Sup. fig. 5). The modified model was developed by converting the original DV3-SS model into a superposition model. The model has initial heads of 0 m, which are conceptualized as no water-level change from modern conditions. Simulated water-level changes are relative to modern heads, where the modern (predevelopment) head distribution was obtained from the calibrated DV3-SS model (Halford and Jackson, 2020).

Death Valley and Franklin Lake remain specified head boundaries (Sup. fig. 4), except that specified heads were changed from predevelopment water-level altitudes to 0 m (Sup. fig. 5). Discharge from springs and evapotranspiration areas were changed from specified-flow rates (well package in MODFLOW; Sup. fig. 4) to head-dependent boundaries in the superposition model (Sup. fig. 5). Head-dependent boundaries were simulated using the drain package in MODFLOW (Harbaugh, 2005). Groundwater discharges from the aquifer if the simulated head is greater than the drain elevation, whereas no discharge occurs if the simulated head is lower than the drain elevation. The drain elevation was assigned equal to the modern water table at 0 m for modern springs and evapotranspiration areas.

To ensure all potential paleo-groundwater-discharge areas were simulated, a head-dependent boundary was specified with a drain for every cell across the entire top surface of the model domain that did not have another boundary condition within the same model cell (Sup. fig. 5). The specified drain elevation for these potential discharge areas was equal to the difference between land-surface altitude and the simulated, modern water-table altitude from the calibrated DV3-SS model. Essentially, the specified drain elevations are depths to water, where groundwater discharge occurs at these model cells if the simulated recharge causes the simulated head to exceed land surface.

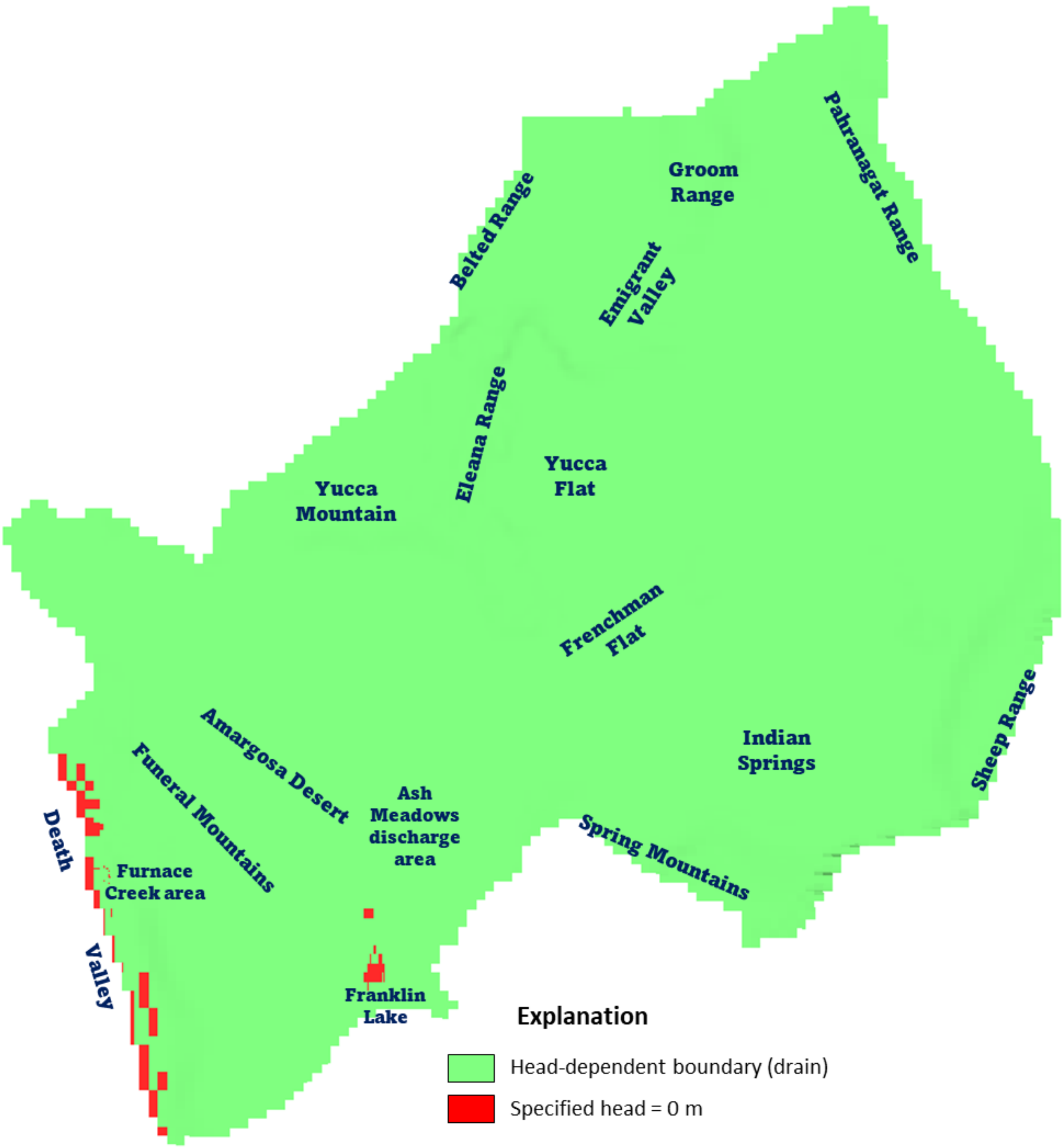

Supplementary figure 5. Domain extent and boundary conditions in the modified DV3-SS model.

Supplementary Discussion

The modified DV3-SS model simulates conditions during past glacial and interglacial periods by assuming that equilibration to steady-state conditions is achieved during each glacial and interglacial period. Steady-state assumption validity was tested by running the DV3-SS model as a transient model for different recharge conditions. The time for Devils Hole water levels to reach near equilibration to a steady-state condition is within 1,000 years.

Transient model simulations were run to determine approximate timescales for equilibration to steady-state conditions. During equilibration from one recharge state to another (e.g., modern to 20% decrease in recharge), water-level changes follow an exponential trend. A large part (80–90%) of the water-level change during equilibration occurs within the first 1,000 years, and the long exponential equilibration timescale (between 5,000 and 10,000 years) occurs for full equilibration to small water-level changes of less than 0.1 m at Devils Hole. More importantly, the transient model that was used to estimate a timescale for equilibration to steady-state conditions simulated instantaneous changes in recharge, whereas natural climate fluctuations result in geologically abrupt (but in reality, very gradual) recharge changes with time. Simulating instantaneous recharge changes assumes that the recharge change occurs all at once. The Devils Hole paleo-water-table record (Wendt et al., 2018) shows that the "abrupt change" in water levels during the last deglaciation occurred over thousands of years. These water levels did not decline for thousands of years because water levels took this long to equilibrate to an instantaneous recharge change. Rather, the elapsed time for recharge to change from a wet glacial climate to a warm interglacial climate was thousands of years. Because changes in recharge span thousands of years, water-level equilibration to these changes can, more-or-less, stay in sync. Therefore, the timescale for water-level equilibration to different climate states in the numerical model is consistent with Devils Hole paleo-water-table data. The steady-state model approach is reasonable because the transient water-level response is short relative to the recharge-change time scale. Furthermore, regardless of whether the model is run as steady-state or transient that equilibrates to steady-state, the end result of the simulated Devils Hole water level is the same.

As stated previously, the modified model was developed by converting the original DV3-SS model into a superposition model. The model has initial heads of 0 m, which are conceptualized as no water-level change from modern conditions. Simulated water-level changes are relative to modern heads, where the modern (predevelopment) head distribution was obtained from the calibrated DV3-SS model (Halford and Jackson, 2020). For example, a glacial scenario was simulated that indicated a 244% increase in recharge from modern conditions was required to induce a 9.5-m rise in Devils Hole water levels, relative to modern (0 m). The simulated water-level change, relative to modern heads, is shown in Sup. fig. 6. As demonstrated in Sup. fig. 6, the simulated head distribution is not an absolute water-level altitude map, but a map of head change from modern conditions.

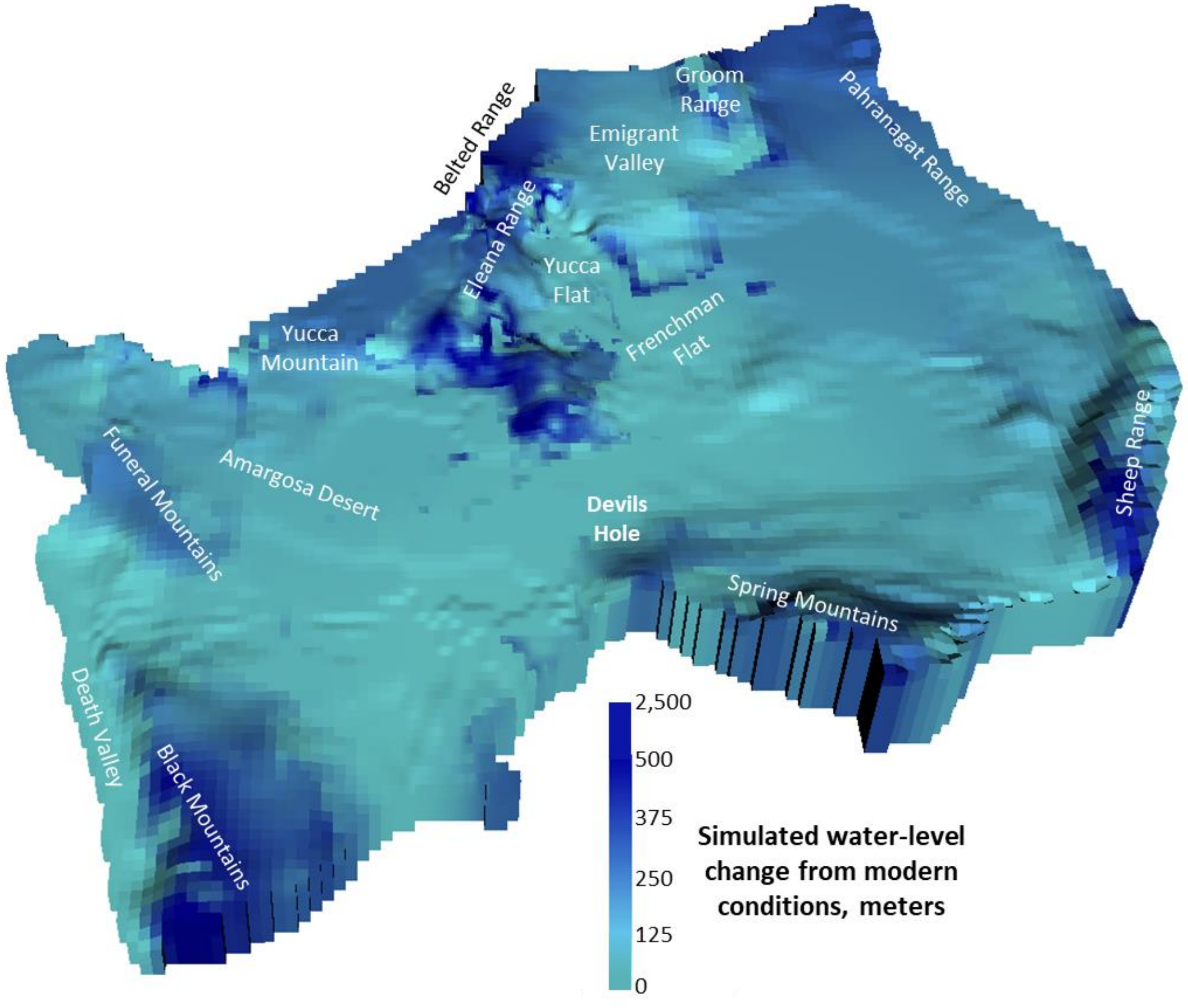

Supplementary figure 6. Glacial scenario simulating a 9.5-m rise in Devils Hole, relative to modern heads at 0 m.

If heads are zero through all model layers (layers 1–6), then this means that the heads have not changed from modern heads. Low-permeability rocks occur in the highland areas of the Belted, Groom, Eleana, and Sheep Ranges and in the Black and Spring Mountains (Sup. fig. 3). In these low-permeability highland areas, recharge rates in the glacial (+9.5 m) scenario exceed the hydraulic conductivity of saturated rocks, which is expected to cause large head changes. Therefore, the head-change distribution in Sup. fig. 6 follows expectations of steep hydraulic gradients in highland recharge areas with low-permeability rocks and gentle hydraulic gradients in more permeable areas.

Simulated recharge distributions for four model scenarios are provided in Sup. figs. 7–10. Most recharge in the glacial (+9.5 m relative to modern level) and interglacial (-1.6 m relative to model level) scenarios occurs in the Spring Mountains and Sheep Range (Sup. figs. 7–8) using the scaled-recharge approach. This result follows expectations because the modern recharge distribution also has most recharge in the Ash Meadows basin occurring from the Spring Mountains and Sheep Range (Halford and Jackson, 2020). Notice that the spatial recharge distributions are similar, but the recharge-rate magnitudes differ, because of the scaling used to generate the model results. The uniform-recharge glacial (+9.5 m relative to modern level) scenario has "smeared" the recharge from the highland areas into the valley floors (Sup. fig. 9), which results in a larger paleo-discharge area footprint in Ash Meadows discharge area (see Figure 4C of main paper). The uniform-recharge interglacial (-1.6 m relative to modern level) scenario reduces low-land recharge significantly (Sup. fig. 10), compared to the scaled-recharge interglacial scenario (Sup. fig. 8).

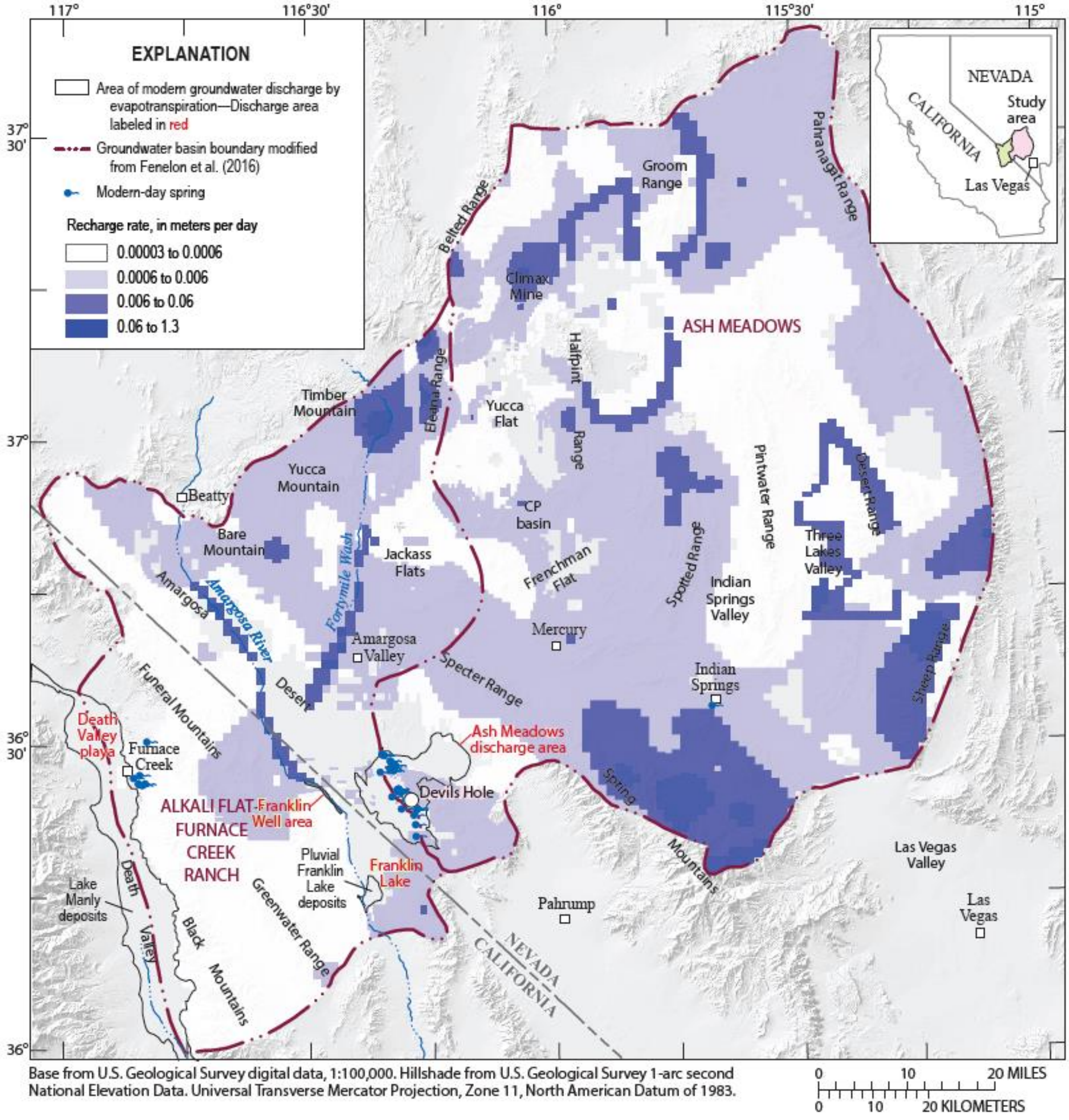

Supplementary figure 7. Simulated recharge distribution for glacial scenario (+9.5 m relative to modern level) using scaled-recharge approach.

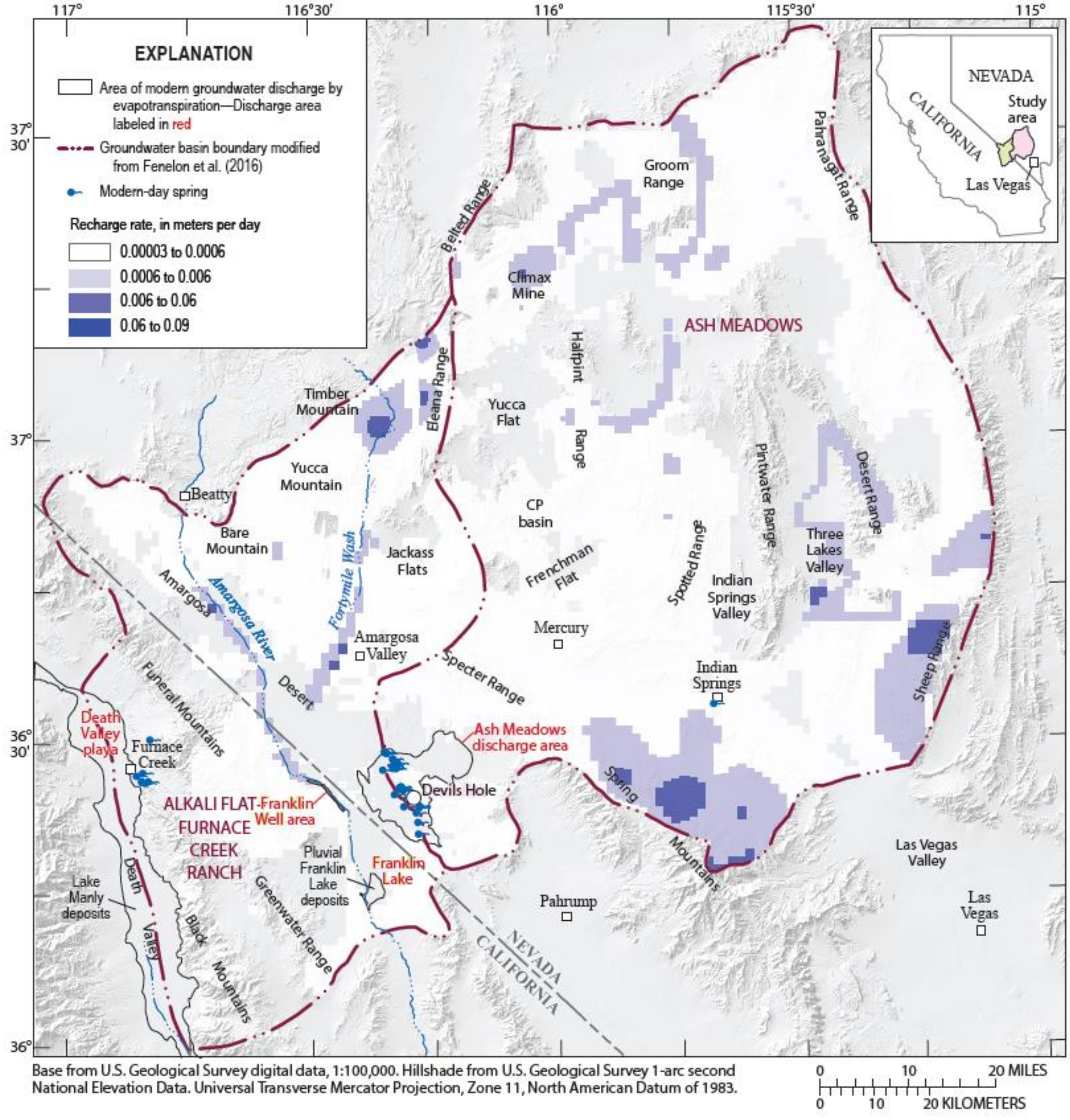

Supplementary figure 8. Simulated recharge distribution for interglacial scenario (-1.6 m relative to modern level) using scaled-recharge approach.

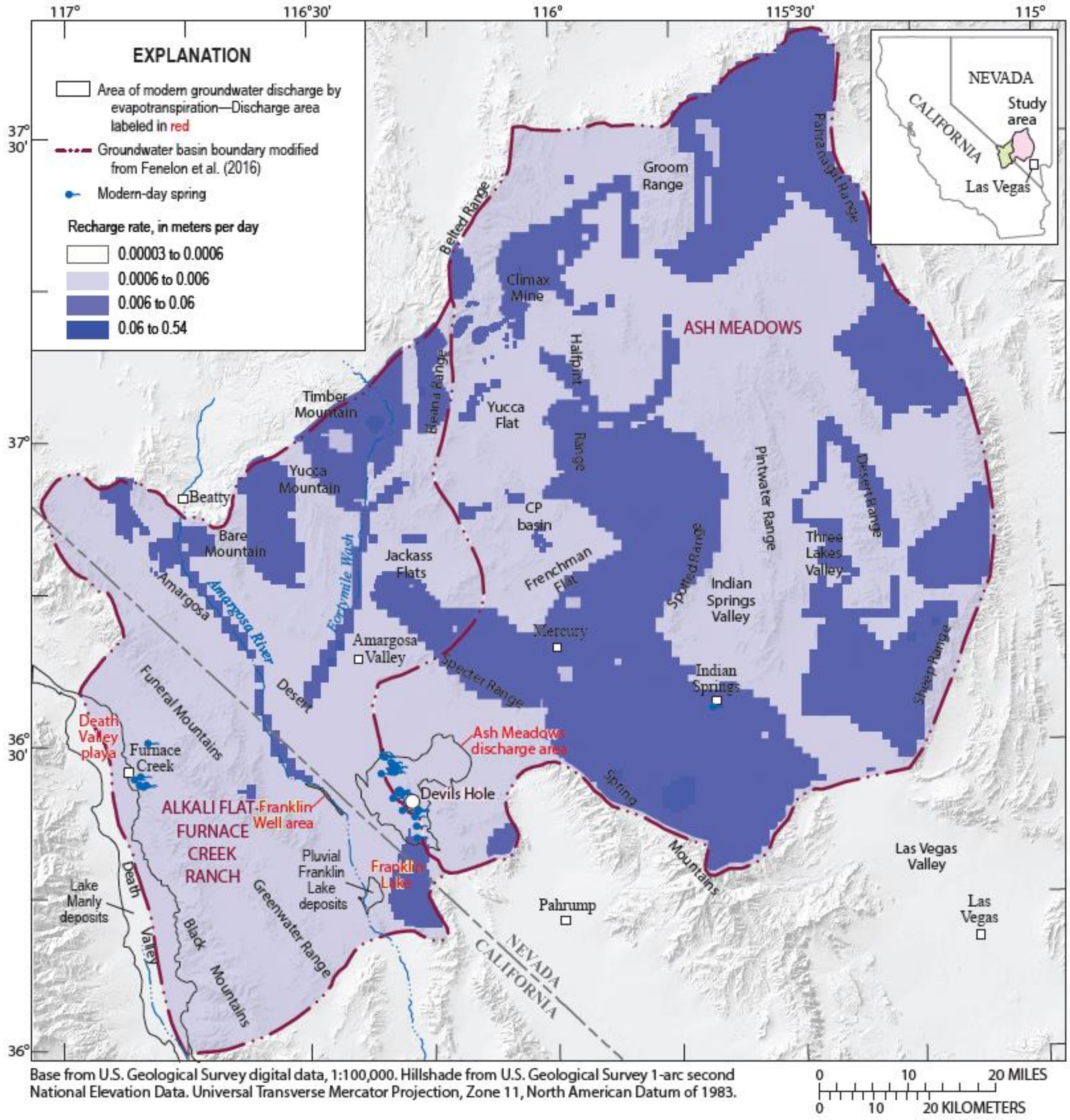

Supplementary figure 9. Simulated recharge distribution for glacial scenario (+9.5 m relative to modern level) using uniform-recharge approach.

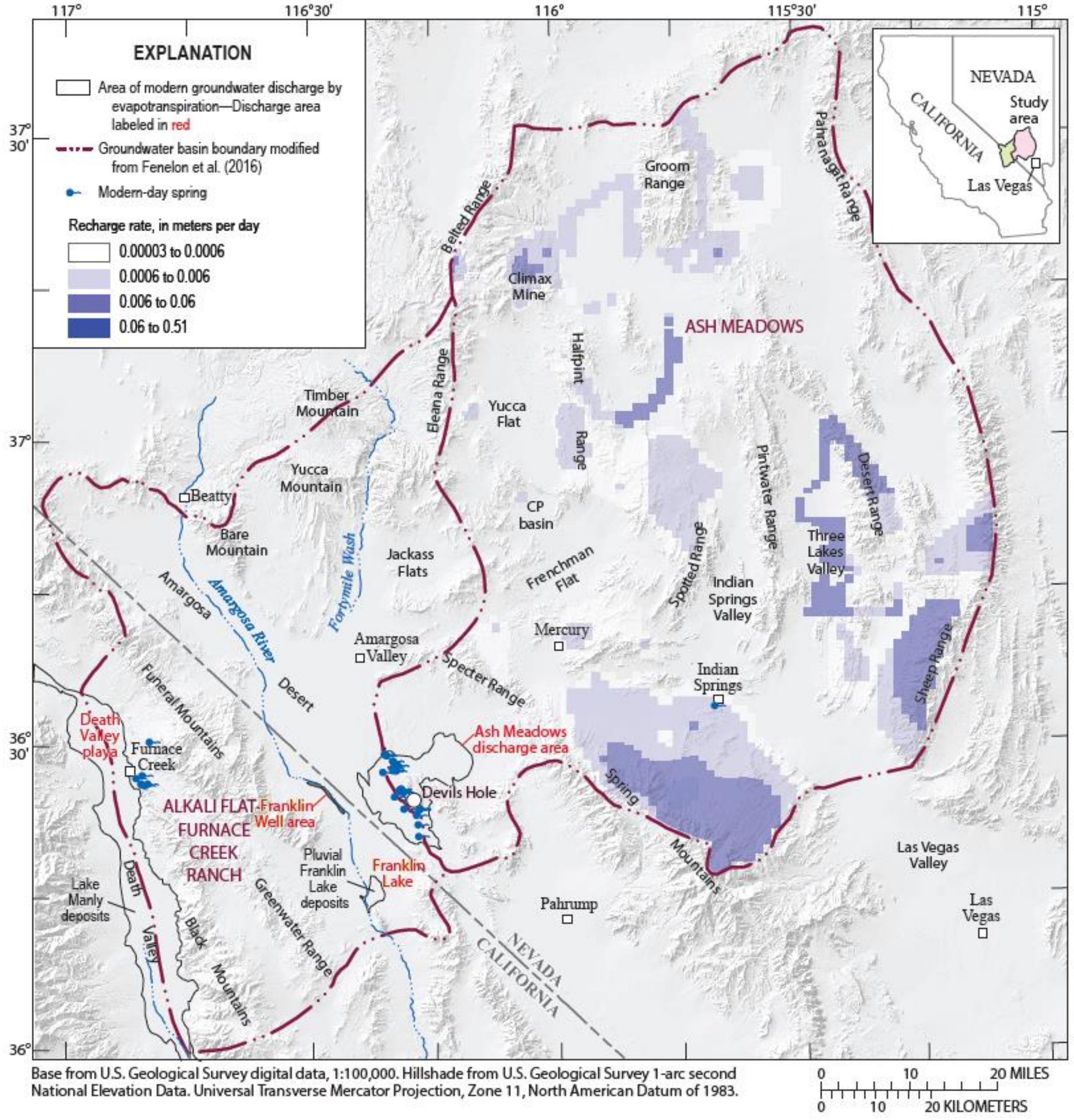

Supplementary figure 10. Simulated recharge distribution for interglacial scenario (-1.6 m relative to modern level) using uniform-recharge approach.

Supplementary References

Bechtel Nevada. A hydrostratigraphic model and alternatives for the groundwater flow and contaminant transport model of Corrective Action Unit 97—Yucca Flat–Climax Mine, Lincoln and Nye Counties, Nevada. U.S. Department of Energy Report DOE/NV/11718-1119. *https://www.osti.gov/biblio/878649* (2006).

Belcher, W. R., Sweetkind, D. S. & Elliott, P. E. Probability distributions of hydraulic conductivity for the hydrogeologic units of the Death Valley regional ground-water flow system, Nevada and California. U.S. Geological Survey Water-Resources Investigations Report 2002–4212. https://doi.org/10.3133/wri024212 (2002).

Blankennagel, R. K. & Weir, J. E., Jr. Geohydrology of the eastern part of Pahute Mesa, Nevada Test Site, Nye, County, Nevada. U.S. Geological Survey Professional Paper 712-B. https://doi.org/10.3133/pp712B (1973).

Byers, F. M. *et al.* Volcanic suites and related cauldrons of the Timber Mountain-Oasis Valley caldera complex, southern Nevada. U.S. Geological Survey Professional Paper 919. https://doi.org/10.3133/pp919 (1976).

Christiansen, R. L. *et al.* Timber Mountain-Oasis Valley caldera complex of southern Nevada. Geol. Soc. Am. Bull. **88**, 7, https://doi.org/10.1130/0016-7606(1977)88<943:TMVCCO>2.0.CO;2 (1977).

Doherty, J. PEST, Model-independent parameter estimation—User manual (5th ed., with slight additions). Brisbane, Australia, Watermark Numerical Computing. (2010).

Fenelon, J. M., Halford, K. J. & Moreo, M. T. Delineation of the Pahute Mesa–Oasis Valley groundwater basin, Nevada (ver. 1.1, May 2016). U.S. Geological Survey Scientific Investigations Report 2015–5175. *https://doi.org/10.3133/sir20155175* (2016).

Fenelon, J. M., Sweetkind, D. S., Elliott, P. E. & Laczniak, R. J. Conceptualization of the predevelopment groundwater flow system and transient water-level responses in Yucca Flat, Nevada National Security Site, Nevada. U.S. Geological Survey Scientific Investigations Report 2012-5196, *https://doi.org/10.3133/sir20125196* (2012).

Fenelon, J. M., Sweetkind, D. S. & Laczniak, R. J. Groundwater flow systems at the Nevada Test Site, Nevada—A synthesis of potentiometric contours, hydrostratigraphy, and geologic structures. U.S. Geological Survey Professional Paper 1771. *https://pubs.usgs.gov/pp/1771/* (2010).

Halford, K. J. & Jackson, T. R. Groundwater characterization and effects of pumping in the Death Valley regional groundwater flow system, Nevada and California, with special reference to Devils Hole. U.S. Geological Survey Professional Paper 1863. *https://doi.org/10.3133/pp1863* (2020).

Harbaugh, A. W. MODFLOW-2005, the U.S. Geological Survey modular ground-water model—The ground-water flow process: U.S. Geological Survey Techniques and Methods, book 6, chap. A16. https://doi.org/10.3133/tm6A16 (2005).

Jackson, T. R. MODFLOW-2005 model used to estimate paleo-recharge volumes in Ash Meadows groundwater basin, southweset Nevada, based on Devils Hole paleo-water-table record. U.S. Geological Survey data release; *https://doi.org/10.5066/P98YZC5P* (2022).

Jackson, T. R., Fenelon, J. M., & Paylor, R. L. Groundwater flow conceptualization of the Pahute Mesa–Oasis Valley Groundwater Basin, Nevada—A synthesis of geologic, hydrologic, hydraulic-property, and tritium data. U.S. Geological Survey Scientific Investigations Report 2020–5134, 100 p., https://doi.org/ 10.3133/ sir20205134 (2021).

Laczniak, R. J., Cole, J. C., Sawyer, D. A. & Trudeau, D. A. Summary of hydrogeologic controls on ground-water flow at the Nevada Test Site, Nye County, Nevada. U.S. Geological Survey Water-Resources Investigations Report 96-4109. *https://doi.org/10.3133/wri964109* (1996).

Noble, D. C. *et al.* Thirsty Canyon tuff of Nye and Esmeralda Counties, Nevada, Article 126—Short papers in geology and hydrology. U.S. Geological Survey Professional Paper 475-D. (1964).

Noble, D. C. *et al.* Stratigraphic relations and source areas of ash-flow sheets of the Black Mountain and Stonewall Mountain volcanic centers, Nevada. J. Geophys. Res. **89**, 8593–8602; https://doi.org/10.1029/JB089iB10p08593 (1984).

Prothro, L. B. & Drellack, S. L., Jr. Nature and extent of lava-flow aquifers beneath Pahute Mesa, Nevada Test Site. U.S. Department of Energy Report DOE/NV/11718-156. https://www.osti.gov/biblio/653925 (1997).

RamaRao, B. S., de Marsily, G. & Marietta, M. G. Pilot point methodology for automated calibration of an ensemble of conditionally simulated transmissivity fields—1, Theory and computational experiments. Water Resour. Res. **31**, 3, 475–493. https://doi.org/10.1029/94WR02258 (1995).

Sawyer, D. A. *et al.* Episodic caldera volcanism in the Miocene southwestern Nevada volcanic field—Revised stratigraphic framework, 40Ar/39Ar geochronology, and implications for magmatism and extension. Geol. Soc. Am. Bull. **106**, 10, 1304–1318. https://doi.org/10.1130/0016-7606(1994)106<1304:ECVITM>2.3.CO;2 (1994).

Simonds, F. W. Geology and hydrothermal alteration at the Calico Hills, Nye County, Nevada. Boulder, University of Colorado, unpublished M.S. thesis. (1989).

Slate, J. L. *et al.* Digital geologic map of the Nevada Test Site and vicinity, Nye, Lincoln, and Clark Counties, Nevada, and Inyo County, California. U.S. Geological Survey Open-File Report 99–554A. scale 1:120,000. https://pubs.usgs.gov/of/1999/ofr-99-0554/ (2000).

Sweetkind, D. S. *et al.* Geology and hydrogeology, chap. B. In: Belcher, W. R., and Sweetkind, D. S. (Eds.), Death Valley regional ground-water flow system, Nevada and California—Hydrogeologic framework and transient ground-water flow model. U.S. Geological Survey Professional Paper 1711. https://doi.org/10.3133/pp1711 (2010).

Taylor, E. M. & Sweetkind, D. S. Three-dimensional geologic mapping of the Cenozoic basin fill, Amargosa Desert basin, Nevada and California. U.S. Geological Survey Scientific Investigations Report 2014–5003. http://dx.doi.org/10.3133/sir20145003 (2014).

Wells, S. G., McFadden, L. D., Renault, C. E. & Crowe, B. M. Geomorphic assessment of late Quaternary volcanism in the Yucca Mountain area, southern Nevada—Implications for the proposed high-level radioactive waste repository. Geology, **18**, 6, 549–553. https://doi.org/10.1130/0091-7613(1990)018<0549:GAOLQV>2.3.CO;2. (1990).

Winograd, I. J. & Thordarson, William. Hydrogeologic and hydrochemical framework, south-central Great Basin, Nevada-California, with special reference to the Nevada Test Site. U.S. Geological Survey Professional Paper 712-C. 126 p. (1975).
